# Actin Cytoskeleton Affects Schwann Cell Migration and Peripheral Nerve Regeneration

**DOI:** 10.3389/fphys.2018.00023

**Published:** 2018-01-25

**Authors:** Yaxian Wang, Qianqian Shan, Jiacheng Pan, Sheng Yi

**Affiliations:** ^1^Key Laboratory of Neuroregeneration of Jiangsu and Ministry of Education, Co-innovation Center of Neuroregeneration, Nantong University, Nantong, China; ^2^Department of Radiotherapy and Oncology, The Affiliated Hospital of Nantong University, Nantong, China

**Keywords:** peripheral nerve injury, RNA deep sequencing, Ingenuity pathway analysis, actin cytoskeleton, Rho, cytochalasin D

## Abstract

Actin cytoskeleton regulates many essential biological functions, including cellular development, shape, polarity, and motility. The organization of actin cytoskeleton has also been associated with numerous physiological and pathological conditions, for instance, the elongation of axonal growth cone during peripheral nerve regeneration. However, the spatio-temporal expression patterns of actin cytoskeleton-related genes and the specific roles of actin cytoskeleton following peripheral nerve injury have not been fully revealed. To address this question, we made rat sciatic nerve crush surgery, collected injured sciatic nerve stumps, analyzed RNA deep sequencing outcomes, and specifically studied two significantly involved canonical pathways that were related with actin, actin cytoskeleton signaling and regulation of actin-based motility by Rho. By using bioinformatic tools and qRT-PCR, We identified and validated differentially expressed genes in these two signaling pathways. Moreover, by applying actin polymerization inhibitor cytochalasin D to sciatic nerve crushed rats, we studied the *in vivo* effect of cytochalasin D and demonstrated that inhibiting actin polymerization would delay the migration of Schwann cells and hinder the repair and regeneration of injured peripheral nerves. Overall, our data revealed the changes of actin cytoskeleton-related genes following peripheral nerve injury and stated the importance of actin cytoskeleton during peripheral nerve regeneration.

## Introduction

Actin is an abundant protein that can be found in essentially all eukaryotic cells. There exist two forms of actin, G-actin, the globular monomeric form, and F-actin, the filamentous polymeric form. Generally, these two forms of actin present in a dynamic balanced state. However, their equilibrium can be disturbed. G-actin can aggregate to F-actin or disaggregate from F-actin and therefore, the actin microfilament can be dynamically regulated (Vavylonis et al., 2005; Reisler and Egelman, 2007). Several specific proteins, for example, the small regulatory GTP-binding proteins, in particular Cdc42, Rac1, and RhoA in the Rho family, can switch the balance between G-actin and F-actin and thus affect the remodeling and organization of actin cytoskeleton. Therefore, these small GTPases can mediate actin cytoskeleton dynamics by affecting the polymerization, branching, and bundling of actin proteins (Ridley and Hall, 1992; Machesky and Hall, 1997; Hall, 1998). It is the precise regulation of actin filament assembly/disassembly that mediates the organization of actin cytoskeleton, affects cellular morphology, adhesion, remodeling, and movement, and contributes to tissue development and wound healing (Pollard and Borisy, 2003).

Additionally, actin cytoskeleton has also been reported to be involved in the process of peripheral nerve repair and regeneration (Kun et al., 2012). Morphological and molecular biological studies suggested that peripheral nerve repair and regeneration involves a series of complicated processes including the degeneration of distal and proximal nerve stumps, the clearance of axon and myelin debris, the formation of the bands of Bungner, and the regrowth and regeneration of injured axon (Fu and Gordon, 1997; Chen et al., 2007; Jones et al., 2016). Among these biological processes, the key step, that is the extension and directional elongation of axon, is largely commanded by the polymerization of actin within the axonal growth cone (Hall, 1998; Dent and Gertler, 2003; Bradke et al., 2012).

Although the importance of actin in peripheral nerve regeneration has been identified, our understanding of role of actin cytoskeleton has not reached the molecular level. The temporal expression patterns and specific roles of actin and actin cytoskeleton-related genes during peripheral nerve regeneration remain largely undetermined. To obtain a better understanding of the dynamics changes of actin cytoskeleton-related genes during peripheral nerve regeneration, in the current study, we used rat sciatic nerve crush model, analyzed previous obtained RNA deep sequencing data (Yi et al., 2015), and specifically investigated dynamic changes in actin cytoskeleton in the injured nerve stumps. Moreover, we applied cytochalasin D, an actin polymerization inhibitor (Cooper, 1987), in Sprague-Dawley (SD) rats to further determine the role of actin cytoskeleton. Our outcomes revealed the significance of actin cytoskeleton-related signaling pathways, identified and validated differentially expressed genes in these signaling pathways, and demonstrated the negative effect of actin polymerization inhibitor on peripheral nerve regeneration.

## Materials and methods

### Animal surgery

Animal surgery was performed as previously described (Yi et al., 2015). Briefly, adult male SD rats were anesthetized intraperitoneally with a mixture of 85 mg/kg trichloroacetaldehyde monohydrate, 42 mg/kg magnesium sulfate, and 17 mg/kg sodium pentobarbital. A skin incision was cut in the left outer mid-thigh and the sciatic nerve was exposed, lifted, and crushed with a sterilized forceps for three times with 10 s for each time. At 1, 4, 7, and 14 days after surgery, rats were sacrificed by decapitation. Rats in the control group (designated as 0 day) were sham-operated and immediately sacrificed. All animal surgery procedures were approved by the Administration Committee of Experimental Animals, Jiangsu, China and performed in accordance with the rules of the Experimental Animal Center of Nantong University.

### Sciatic nerve stump collection and RNA isolation

Rat sciatic nerve stumps of 5 mm in length were harvested and stored at −80°C for subsequent isolation. Total RNAs were isolated by using Trizol Reagent (Life technologies, Carlsbed, CA, USA) according to the manufacturer's instructions. RNeasy spin columns (Qiagen, Valencia, CA, USA) were applied to eliminate contaminating DNAs. NanoDrop ND-1000 spectrophotometer (Infinigen Biotechnology Inc., City of Industry, CA, USA) was used to measure the concentrations of isolated RNAs.

### RNA deep sequencing and bioinformatic analysis

RNA deep sequencing was previously performed by using Illumina HiSeq^TM^ 2000 (Yi et al., 2015). Sequencing data were normalized by using reads per kilobase transcriptome per million mapped reads (RPKM) method (Mortazavi et al., 2008) and the expression levels of RNAs at 1, 4, 7, and 14 days following sciatic nerve crush were compared with the expression levels at 0 day. RNAs with a fold change greater than 2 or less than −2 and a false discover rate (FDR) less than 0.001 were considered as differentially expressed genes.

Differentially expressed genes were analyzed by using Core Analysis function in Qiagen's Online IPA software program (Ingenuity Systems Inc., Redwood City, CA, USA) to interpret enriched canonical signaling pathways. The *p*-values of two actin cytoskeleton-related canonical signaling pathways, actin cytoskeleton signaling and regulation of actin-based motility by Rho, were calculated by the Fisher Exact test right-tailed according to the IPA manual (http://ingenuity.force.com/ipa/IPATutorials?id=kA250000000TN3fCAG). Differentially expressed genes in these two canonical signaling pathways were further analyzed and illustrated by using the Venny 2.1.0 online software (http://bioinfogp.cnb.csic.es/tools/venny/index.html) (Oliveros, 2007). Heatmap and hierarchical clustering were also generated to demonstrate the temporal expression patterns of actin cytoskeleton-related genes.

### qRT-PCR

qRT-PCR was performed as previously described (Yi et al., 2015) to validate the accuracy of RNA deep sequencing. Briefly, isolated RNAs were reverse transcribed to cDNA by using the Prime-Script reagent Kit (TaKaRa, Dalian, China) and qRT-PCR was performed by using SYBR Green Premix Ex Taq (TaKaRa) on a StepOne Real-time PCR System (Applied Biosystems, Foster City, CA, USA). Relative quantification of target genes was performed by using the comparative 2^−ΔΔ*Ct*^ method with 18s as the reference gene. The sequences of primer pairs were listed in Supplementary Material S1.

### *In vivo* experiments

Cytochalasin D was dissolved in DMSO to a high concentration storage solution and diluted to a final concentration of 400 or 1,000 nM by using saline before use. The final content of DMSO was 0.1%. Adult SD rats were equally and randomly divided into three groups with six rats in each group to receive the injection of 400 nM cytochalasin D, 1,000 nM cytochalasin D, or saline containing 0.1% DMSO (considered as the DMSO group). These rats underwent sciatic nerve crush as previously described. After rat sciatic nerve injury, 6 μl of 400 or 1,000 nM cytochalasin D or saline containing 0.1% DMSO was directly injected into the epineurium in the crush stump by using a microsyringe. The point of the microsyringe was left for 1 min in the injection site to prevent liquid outflow.

### Immunohistochemistry staining

Immunohistochemistry staining was performed at 3 or 5 days following nerve crush and cytochalasin D injection. Rat sciatic nerve tissues were collected onto microscope slides, fixed in 4% paraformaldehyde for 15 min, washed in PBS, and subjected to goat serum incubation. After blocking with 5% goat serum for 30 min, sciatic nerve slices were incubated with rabbit anti-SCG10 (Novus Biologicals, Littleton, CO, USA) primary antibody or rabbit anti-S100β (Sigma, St. Louis, MO, USA) and mouse anti-NF-H (Sigma) overnight at 4°C. Secondary immunofluorescent labeling was performed by using goat anti-rabbit cy3 (1:1,000; Abcam) or goat anti-mouse 488 (1:500; Abcam) for 2 h at room temperature. Immunohistochemistry images were taken under fluorescence microscopy (Axio Imager M2, Carl Zeiss Microscopy GmbH, Jena, Germany).

### Compound muscle action potential (CMAP) recording

CMAP responses were determined as previously described (Wang et al., 2015) at 4 weeks after sciatic nerve injury. Briefly, rats were anesthetized, their injured sciatic nerve stumps were re-exposed, electrodes were inserted into the mid-belly of gastrocnemius, and electrical stimuli were applied to the proximal and distal nerve stumps. The amplitudes of CMAP recordings were measured on the belly of target gastrocnemius muscle and recorded.

### Data analysis

All numerical results were reported as means ± SEM. Statistical analysis and graphs were performed by using GraphPad Prism 6.0 (GraphPad Software, Inc., La Jolla, CA, USA). Differences between treatment groups and the control group were tested by using one-way analysis of variance (ANOVA) test with *post-hoc* contrasts by Dunnett's multiple comparisons test. A *p*-value less than 0.05 was considered as significant different.

## Results

### Involvement of actin-related signaling pathways following sciatic nerve injury

Previous obtained RNA deep sequencing data were subjected to IPA canonical signaling pathway analysis to calculate the *p*-value of actin cytoskeleton-related signaling pathways and to identify the significantly these signaling pathways. Two actin cytoskeleton-related signaling pathways, actin cytoskeleton signaling and regulation of actin-based motility by Rho, exhibited a significant *p*-value less than 0.05 following sciatic nerve crush (Yi et al., 2015). These two signaling pathways and the differentially expressed genes in these two signaling pathways were further investigated. IPA canonical pathway analysis revealed that at 1 day after sciatic nerve crush, actin cytoskeleton signaling had a *p*-value of 10^−5.49^. At this time point, a total number of 75 genes were differentially expressed. Actin cytoskeleton signaling remained significantly involved at later points, with a *p*-value of 10^−4.86^, 10^−2.71^, and 10^−2.37^ and 72, 49, and 38 differentially expressed genes at 4, 7, and 14 days after nerve crush, respectively. Similarly, signaling pathway regulation of actin-based motility by Rho was also significantly activated following sciatic nerve crush, having a p-value of 10^−3.18^, 10^−2.97^, and 10^−1.84^, and 10^−1.59^ and affecting 33, 32, 22, and 17 genes at 1, 4, 7, and 14 days after nerve crush, respectively (Table 1).

**Table 1:** Significance of actin cytoskeleton signaling and regulation of actin-based motility by Rho at each time point following sciatic nerve crush.

	**−log(*p*-value)**	**Number of differentially expressed genes**
**ACTIN CYTOSKELETON SIGNALING**		
1 day	5.49	75
4 days	4.86	72
7 days	2.71	49
14 days	2.37	38
**REGULATION OF ACTIN-BASED MOTILITY BY RHO**		
1 day	3.18	33
4 days	2.97	32
7 days	1.84	22
14 days	1.59	17

*The −log (p-value) of these signaling pathways were listed*.

### Analysis of actin cytoskeleton signaling

**Figure 1:**
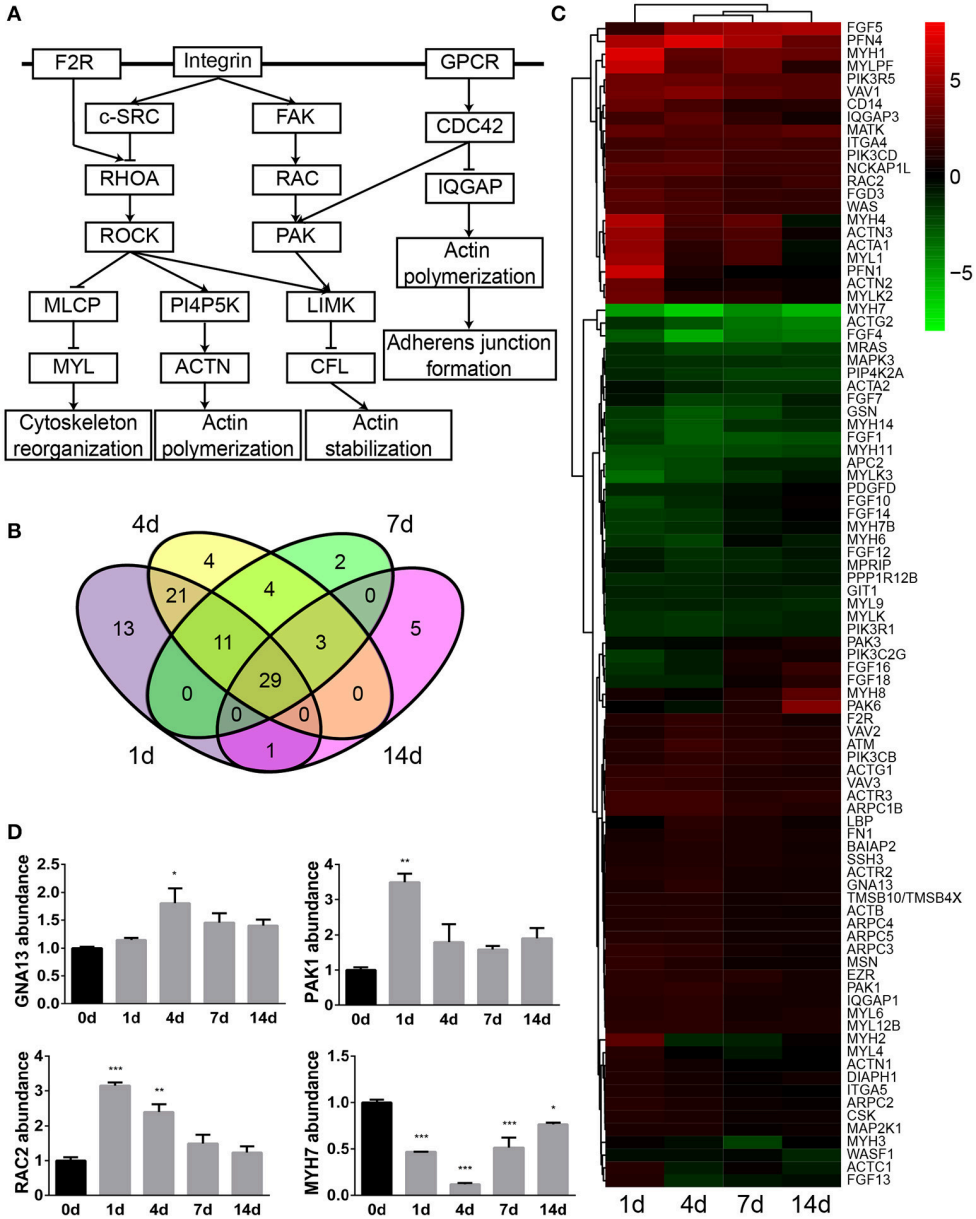
Actin cytoskeleton signaling was significantly involved following sciatic nerve crush. **(A)** The schematic diagram of actin cytoskeleton signaling. The diagram was modified based on IPA database. **(B)** The Venn diagram of differentially expressed genes in actin cytoskeleton signaling. **(C)** The heatmap and hierarchical clustering of differentially expressed genes in actin cytoskeleton signaling. The expression levels of differentially expressed genes were indicated by the color bar. Up-regulated genes were labeled in red while down-regulated genes were labeled in green. **(D)** qRT-PCR analysis of representative genes in actin cytoskeleton signaling. The relative expression levels of GNA13, PAK1, RAC2, and MYH7 were calculated by comparative Ct with reference gene 18s. The expression levels of GNA13, PAK1, RAC2, and MYH7 at 1, 4, 7, and 14 days were compared with their expression levels at 0 day. The asterisks indicated significant different from 0 day: ^*^p < 0.05, ^**^p < 0.01, and ^***^p < 0.001.

The actin cytoskeleton is critical for a variety of dynamic activities such as cellular assembly and organization, cellular function and maintenance, and tissue and organismal growth and development. IPA schematic diagrams of canonical signaling pathway actin cytoskeleton signaling showing the relative gene expression changes at 1, 4, 7, and 14 days following sciatic nerve crush were listed in Supplementary Material S2. To demonstrate central genes in the actin cytoskeleton signaling, a simplified diagram was constructed based on IPA database and showed in Figure 1A. Membrane receptors such as F2R (coagulation factor II (thrombin) receptor) activate RHOA, which binds to and activates Rho kinase ROCK. Activated ROCK increases the phosphorylation of MYL (myosin light chain) by inhibiting MLCP (myosin light chain phosphatases), increases the activity of PI4P5K (phosphatidyl inositol 4-phosphate 5-kinase), inhibits the function of CFL (cofilin), and thus affects the arrangement of the actin cytoskeleton. Integrin, on the other hand, activates c-SRC and inhibits the activation of RHOA. Integrin and GPCR (guanosine-binding protein coupled receptor) also activate PAK (p21-activated kinase) and disengage stress fiber and focal adhesion by activating FAK (focal adhesion kinase) and CDC42, respectively. Activated CDC42 also hinders scaffold protein IQGAP1 (Ras GTPase-activating-like protein) and thus regulates actin cytoskeleton organization, cellular adhesion, and adherens junction formation.

Differentially expressed genes in IPA canonical signaling pathway actin cytoskeleton signaling were then investigated in detail. Venn diagram showed that in actin cytoskeleton signaling, a total number of 93 genes were differentially expressed while 29 genes were commonly dysregulated at 1, 4, 7, and 14 days after sciatic nerve crush (Figure 1B). The relative expression levels of these 93 genes were then studied and listed in Supplementary Material S3. Additionally, the temporal expression patterns of these differentially expressed genes were demonstrated in a heatmap (Figure 1C). The majority of differentially expressed genes in this signaling pathway were kept up-regulated following sciatic nerve crush. Around 1/3 of genes were kept down-regulated while the rest of differentially expressed genes exhibited a curved expression pattern.

The expression levels of GNA13 [guanine nucleotide binding protein (G protein), alpha 13], PAK1, RAC2 [ras-related C3 botulinum toxin substrate 2 (rho family, small GTP binding protein Rac2)], and MYH7 (myosin, heavy chain 7, cardiac muscle, beta), four genes in the axon cytoskeleton signaling pathway, were determined by qRT-PCR. qRT-PCR results suggested that GNA13, PAK1, and RAC2 were up-regulated while MYH7 was down-regulated after sciatic nerve injury (Figure 1D). These observations were in consistent with RNA deep sequencing data, indicating the accuracy and reliability of RNA deep sequencing.

### Analysis of regulation of actin-based motility by Rho

**Figure 2:**
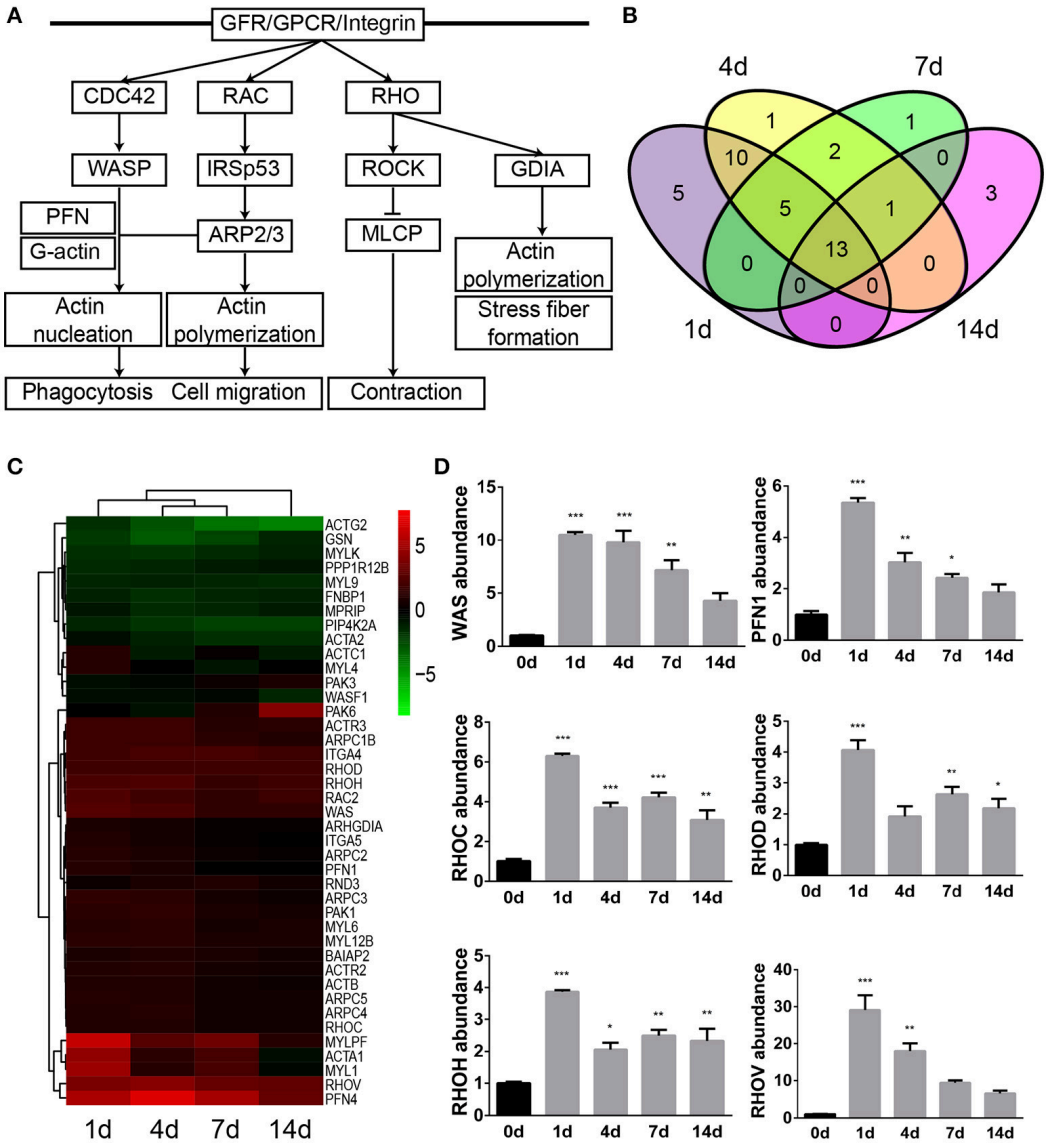
Regulation of actin-based motility by Rho was significantly involved following sciatic nerve crush. **(A)** The schematic diagram of regulation of actin-based motility by Rho. The diagram was modified based on IPA database. **(B)** The Venn diagram of differentially expressed genes in regulation of actin-based motility by Rho. **(C)** The heatmap and hierarchical clustering of differentially expressed genes in regulation of actin-based motility by Rho. The expression levels of differentially expressed genes were indicated by the color bar. Up-regulated genes were labeled in red while down-regulated genes were labeled in green. **(D)** qRT-PCR analysis of representative genes in regulation of actin-based motility by Rho. The relative expression levels of WAS, PFN1, RHOC, RHOD, RHOH, and RHOV were calculated by comparative Ct with reference gene 18s. The expression levels of WAS, PFN1, RHOC, RHOD, RHOH, and RHOV at 1, 4, 7, and 14 days were compared with their expression levels at 0 day. The asterisks indicated significant different from 0 day: ^*^p < 0.05, ^**^p < 0.01, and ^***^p < 0.001.

Previous schematic diagrams of actin cytoskeleton signaling showed that the arrangement and organization of actin was regulated by many signaling cascades, in particular by members of the Rho family of small GTPases. Therefore, another significantly involved IPA canonical signaling pathway that is related to actin, regulation of actin-based motility by Rho, was studied as well. Binding of ligands to receptors such as GFR (growth factor receptor), GPCR, and integrin stimulates the activation of Rho family proteins. Activated CDC42 and RAC activates scaffolding proteins WASP (Wiskott-Aldrich syndrome protein) and IRSp53 (insulin receptor substrate of 53 kDa), respectively, causes the formation of WASP/PFN (profilin)/G-actin/ARP2/3 (actin related protein 2/3 complex) complex, and leads to the nucleation and polymerization of actin, the formation of filopodia, microspike, and lamellipodia, phagocytosis, and cell migration. Likewise, activated RHO binds to and activates ROCK and GDIA (Rab GDP dissociation inhibitor alpha), and thus regulates force-induced contact formation and cell motility (Figure 2A and Supplementary Material S4).

A total number of 41 genes were differentially expressed following sciatic nerve crush (Supplementary Material S5). Among these differentially expressed genes, 5 genes (PFN1, MYL4, ACTC1, ITGA5, and ARHGDIA) were dysregulated exclusively at 1 day after sciatic nerve crush, 1 gene (PFN4) was dysregulated exclusively at 4 days after injury, 1 gene (RND3) was dysregulated exclusively at 7 days after injury, and 3 genes (PAK6, WASF1, and PAK3) were dysregulated exclusively at 14 days after injury. 13 genes were dysregulated at all tested time points following sciatic nerve crush (Figure 2B). The heatmap showed that over 1/2 genes were remain up-regulated, less than 1/4 genes were remain down-regulated, and 5 genes (ACTC1, MYL4, PAK6, ACTA1, and MYL1) exhibited altered temporal expression patterns (Figure 2C).

Similarly, qRT-PCR experiment was performed to validate the temporal expression patterns of several key genes in the regulation of actin-based motility by Rho signaling pathway. Consistent with RNA deep sequencing data, the mRNA abundance of WAS, PFN1, RHOC (ras homolog family member C), RHOD (ras homolog family member D), RHOH (ras homolog family member H), and RHOV (ras homolog family member V) were elevated following sciatic nerve crush (Figure 2D).

### Effects of actin polymerization inhibitor on sciatic nerve regeneration

**Figure 3:**
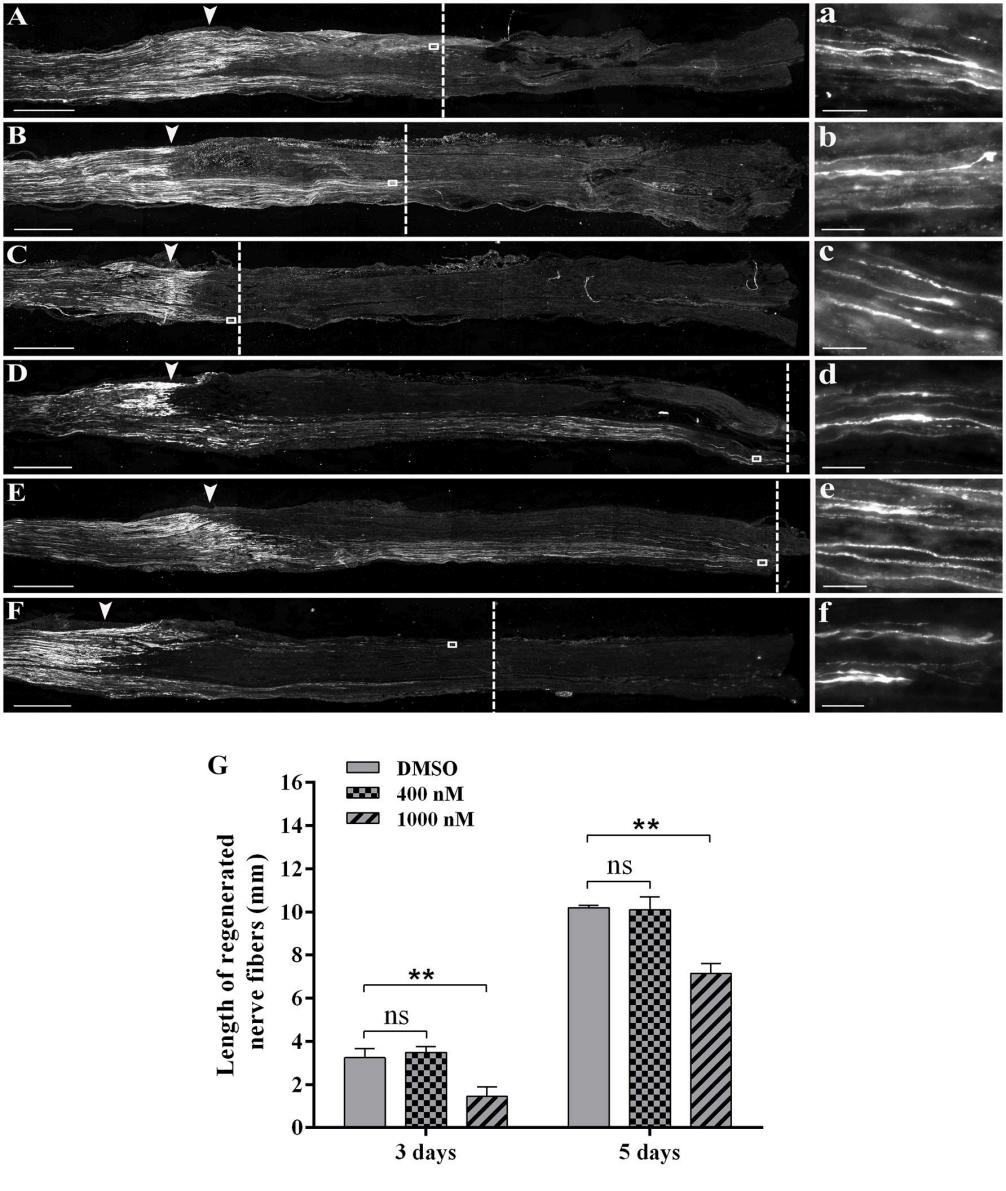
Cytochalasin D treatment slowed axon outgrowth. **(A–C)** Representative immunohistochemistry images with anti-SCG10 of regenerating nerves treated with **(A)** DMSO, **(B)** 400 nM cytochalasin D, and **(C)** 1000 nM cytochalasin D at 3 days after sciatic nerve crush. Arrows indicated injury site. Higher magnifications of boxed areas were shown in (a–c), respectively. **(D–F)** Representative immunohistochemistry images with anti-SCG10 of regenerating nerves treated with **(D)** DMSO, **(E)** 400 nM cytochalasin D, and **(F)** 1000 nM cytochalasin D at 5 days after sciatic nerve crush. Arrows indicated injury site. Higher magnifications of boxed areas were shown in (d–f), respectively. Scale bars represented 1,000 μm **(A–F)** and 20 μm (a–f). **(G)** Summarized results of the length of regenerated nerve fibers from 3 paired experiments. The length of regenerated nerve fibers in 400 nM or 1,000 nM cytochalasin D treated group was compared with the length of regenerated nerve fibers in the DMSO group. The asterisks indicated significant different from DMSO group, ^**^p < 0.01.

Outcomes from bioinformatic analysis showed that actin-related genes and signaling pathways were extensively engaged following peripheral nerve injury. *In vivo* studies were then performed to identify the specific role of actin on peripheral nerve repair and regeneration. Directly after sciatic nerve crush, F-actin polymer inhibitor cytochalasin D was injected at the crush site and immunohistochemistry was performed to determine axonal growth conditions. Immunohistochemistry staining with regenerating axons marker anti-SCG10 showed that at 3 days after crush, axons in the DMSO group regenerated and extended for about 3.5 mm (Figures 3A,G). The length of regenerating fibers in rats treated with 400 nM cytochalasin D was not significantly altered (Figures 3B,G). However, axon regrowth was extensively reduced in rats treated with 1,000 nM cytochalasin D (Figure 3C,G). Similarly, at 5 days after sciatic nerve crush, 400 nM cytochalasin D did not significantly affect axon extension while cytochalasin D at a higher concentration (1,000 nM) robustly inhibited axon growth (Figures 3D–G).

**Figure 4:**
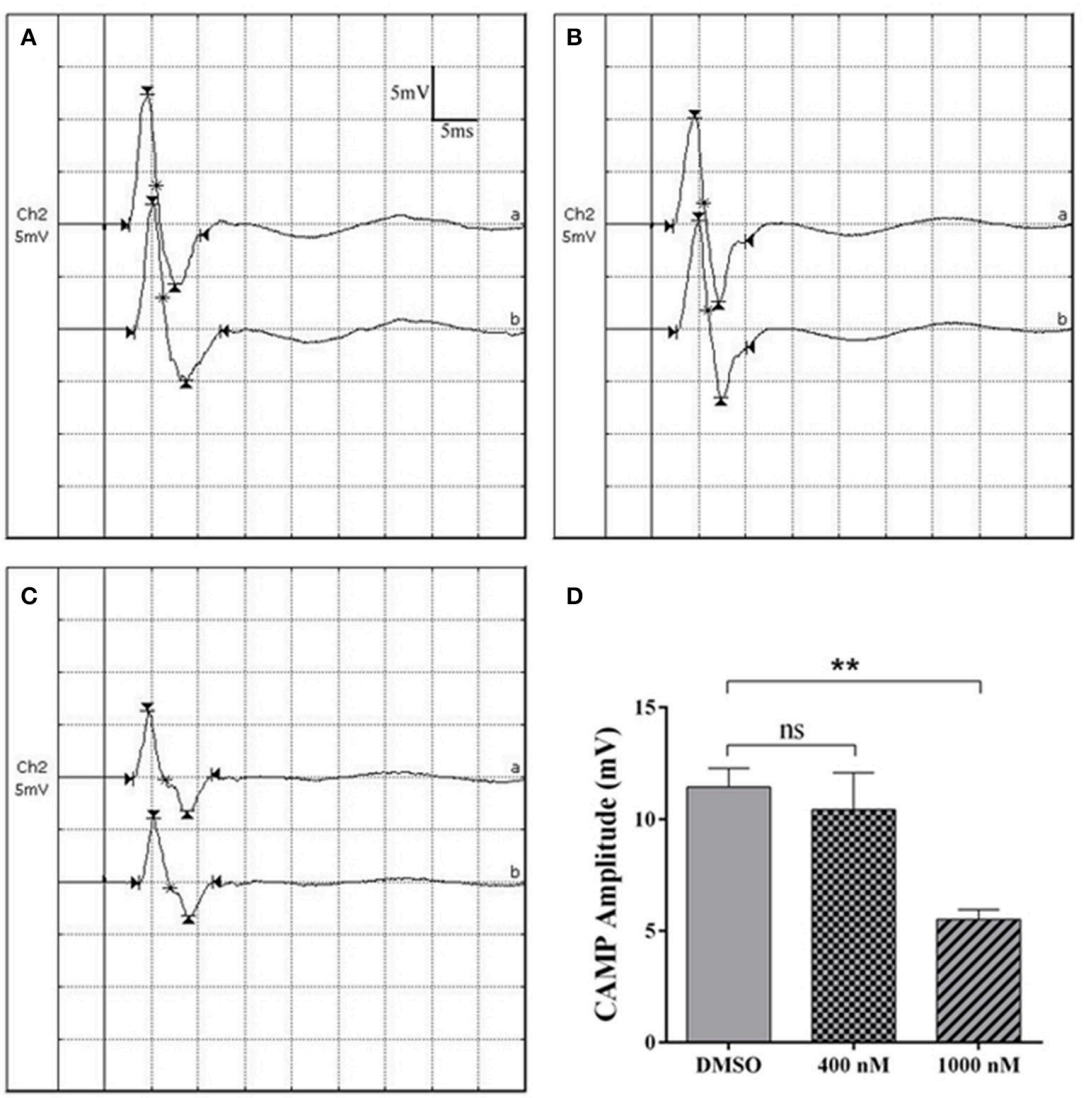
Cytochalasin D treatment decreased CMAP amplitude**. (A–C)** Representative CMAP recordings of rat sciatic nerve treated with **(A)** DMSO, **(B)** 400 nM cytochalasin D, and **(C)** 1,000 nM cytochalasin D at 4 weeks after sciatic nerve crush. **(D)** Summarized results of peak CMAP amplitude from 3 paired experiments. The peak CMAP amplitude in 400 or 1,000 nM cytochalasin D treated group was compared with peak CMAP amplitude in the DMSO group. The asterisks indicated significant different from DMSO group, ^**^p < 0.01.

Electrophysiological parameters were also measured to evaluate the functional recovery of injured sciatic nerve stumps. CMAP recordings showed that at 4 weeks after sciatic nerve crush, in DMSO group, the detected peak amplitude was about 12 mV (Figures 4A,D). Rats received 400 nM cytochalasin D injection had slightly lower CAMP amplitudes (Figures 4B,D) while rats injected with 1,000 nM cytochalasin D exhibited significantly lower CAMP amplitudes (Figures 4C,D). These outcomes suggested that cytochalasin D had an *in vivo* inhibitory effect on nerve regeneration.

### Effects of actin polymerization inhibitor on schwann cell migration

**Figure 5:**
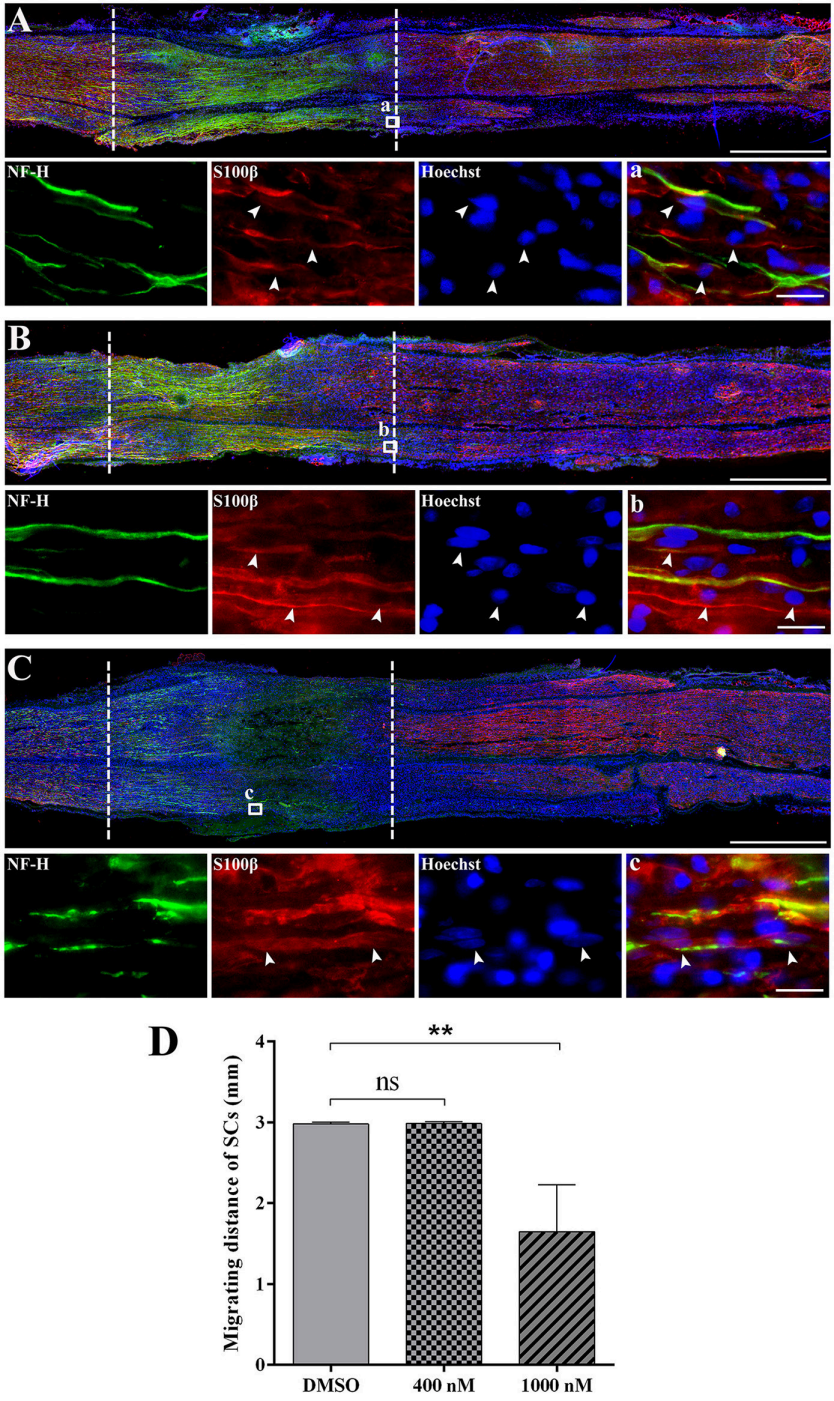
Cytochalasin D treatment disturbed the migration of Schwann cells. **(A–C)** Representative immunohistochemistry images of rat sciatic nerve treated with **(A)** DMSO, **(B)** 400 nM cytochalasin D, and **(C)** 1000 nM cytochalasin D at 3 days after sciatic nerve crush. Areas between the dotted lines indicated injury site. Red indicated S100 staining of Schwann cells, green indicated NF-H staining of axons, and blue indicated Hoechst staining of cell nuclei. Higher magnifications of boxed areas were shown in (a–c), respectively. Arrows indicated the migrated Schwann cells. Scale bars represented 1,000 μm **(A–C)** and 20 μm (a–c). **(D)** Summarized results of the migrating distance of Schwann cells from 3 paired experiments. The migrating distance of Schwann cells (SCs) in 400 or 1,000 nM cytochalasin D treated group at 3 days after sciatic nerve crush was compared with the migrating distance of Schwann cells in the DMSO group. The asterisks indicated significant different from DMSO group, ^**^p < 0.01.

Schwann cell migration largely contributes to peripheral nerve repair since the regrowth of axon requires the guidance of migrated Schwann cells. Therefore, after the injection of cytochalasin D, we examined the localization of Schwann cells to test whether actin polymerization inhibitor would affect Schwann cell migration. Immunohistochemistry staining with Schwann cell marker S100β and axon marker NF-H showed that at 3 days after crush, in the DMSO group and 400 nM cytochalasin D group, the crush segment was completely connected by migrated Schwann cells, a large number of regenerated axons grow through the crushed segment, and some axons even grow to the distal nerve stumps (Figures 5A,B). However, in the 1,000 nM cytochalasin D group, the migration of Schwann cells and the regrowth of axons were largely limited (Figure 5C). Summarized data showed that the distance of migrating Schwann cells was significantly shorter in the 1,000 nM cytochasin D group (Figure 5D). Moreover, the migrated Schwann cells did not fully bridge the crush segment until 5 days after crush (Supplementary Material S6), showing that cytochalasin D repressed Schwann cell migration.

## Discussions

In the current study, we used RNA deep sequencing and IPA bioinformatic analysis to investigate the dynamic changes of genes linked to axon cytoskeleton in the sciatic nerve stumps at 0, 1, 4, 7, and 14 days after sciatic nerve crush. IPA canonical signaling pathway analysis suggested that signaling pathways related with actin cytoskeleton (actin cytoskeleton signaling and regulation of actin-based motility by Rho) were kept significantly activated during the whole process of peripheral nerve repair and regeneration. Heatmap analysis illustrated that many genes in these pathways were continuously dysregulated at all-time points following peripheral nerve injury.

Notably, the temporal expression patterns of axon cytoskeleton-related genes were different from the expression patterns of all differentially expressed genes. The transcriptional profiling of differentially expressed genes in crushed sciatic nerve suggested that the majority of differentially expressed genes were up-regulated while the number of down-regulated genes was robustly smaller (11,752 up-regulated genes and 1,969 down-regulated genes at day 1; 12,119 up-regulated genes and 2,202 down-regulated genes at day 4; 13,390 up-regulated genes and 1,355 down-regulated genes at day 7; and 5,615 up-regulated genes and 1,364 down-regulated genes at day 14 after sciatic nerve crush) (Yi et al., 2015; Qin et al., 2016). However, in canonical signaling pathway actin cytoskeleton signaling, the ratio of down-regulated genes to up-regulated genes was much higher, suggesting that the many genes linked to the integrity of cell skeleton and the maintenance of tissue structure might be down-regulated. These changes in transcriptional expressions were consistent with morphological features that the nerve stumps were disrupted following injury (Hogan, 2008; Knott et al., 2014) and implied the molecular cytoskeletal machinery of peripheral nerve injury.

Canonical signaling pathway regulation of actin-based motility by Rho was also studied in detail since members of the Rho family were essential regulators of the organization and dynamics of the actin cytoskeleton (Hall, 1998; Hall and Nobes, 2000; Raftopoulou and Hall, 2004; McKerracher et al., 2012). Emerging studies have been performed to identify the specific roles of members of the Rho family, specifically Cdc42, Rac1, and RhoA. Activated Cdc42 and Rac would promote the outgrowth of neurite and the motility of the axonal growth cone (Meyer and Feldman, 2002). On the other hand, activated RhoA would inhibit neurite outgrowth and impede the recovery of injured nerves (Madura et al., 2007; Fujita and Yamashita, 2014; Rozés Salvador et al., 2016). Interestingly, RhoA not only affect neurons, but also affect Schwann cells, the unique glial cell in the peripheral nervous system. Lentiviral vector-mediated knock-down of RhoA would hinder Schwann cell proliferation and migration and lead to hypomyelination (Wen et al., 2017). Although the effect of Rho GTPases on neurons and glial cells has been well demonstrated, the temporal expression patterns of members of the Rho family remain unknown. And our current study filled the existing gap by means of big data analysis.

Sequencing data from dorsal root ganglia at 0, 1, 4, and 7 days following sciatic nerve crush (Gong et al., 2016) was analyzed and compared with current observations. Our previously detected gene list identified 93 differentially expressed genes in the sciatic nerve stumps in actin cytoskeleton signaling at 1, 4, 7, and 14 days (Supplementary Material S3). Among these genes, a total of 88 genes were differentially expressed in the sciatic nerve stumps at 1, 4, and 7 days (genes FGF18, MYH8, PAK3, PAK6, and WASF1 were only differentially expressed at 14 days after nerve injury). But only 13 genes (ACTA1, ACTN2, ACTN3, CD14, FGF23, MYH4, MYH7, MYL1, MYLK2, MYLPF, WAS, FN1, and INS1) were differentially expressed in the dorsal root ganglia (Supplementary Material S7). The number of differentially expressed genes in signaling pathway the regulation of actin-based motility by Rho in the dorsal root ganglia was also less. Compared with 38 differentially expressed genes in the sciatic nerve stumps, only 5 genes (ACTA1, MYL1, MYLFP, RHOC, and WAS) were differentially expressed in the dorsal root ganglia at 1, 4, and 7 days following sciatic nerve crush (Supplementary Material S7). The comparison result, from the aspect of gene number, showed that after peripheral nerve injury, the dynamic changes in the sciatic nerve stump was more robust than in the dorsal root ganglia. And it might be possible that many actin cytoskeleton-related changes occurred in Schwann cells and other cells responding to the injured nerve stump other than the neurons.

Furthermore, we performed an *in vivo* study and tested the effect of cytochalasin D, a fungal toxin that binds to F-actin polymer and prevents subsequent addition of G-actin to the existing polymerized filament, on peripheral nerve regeneration. Cytochalasin D has commonly been used to disrupt F-actin polymerization. Pharmacological treatment of Cytochalasin D would induce collapse and retraction of leading front, inhibit some translocation of Schwann cells, and restrain neurite outgrowth of dorsal root ganglia neurons (Wang et al., 2012; Valakh et al., 2015). These outcomes suggested that actin cytoskeleton is critical for the physiological activities of both neurons and glial cells. Our study, on the other hand, directly showed that cytochalasin D could hinder the extension of regenerating axons, the migration of Schwann cells, and the functional recovery of injured nerves. Combining with finding that more genes were changed in the sciatic nerve stump than in the dorsal root ganglia, it might be possible that the disruption of actin cytoskeleton affected the phenotype of Schwann cells, which further led to a less permissive microenvironment for subsequent regeneration.

It is notable that many other cell types in the injured sciatic nerve stump besides Schwann cells, such as macrophages, pericytes, and fibroblasts, may also be affected by the disruption of actin cytoskeleton. For instance, it was demonstrated that cytochalasin D could inhibit phagocytosis (Kankkunen et al., 2010; Zhang et al., 2015). Therefore, Schwann cell and macrophage-mediated phagocytosis of axon and myelin debris might be impaired, Wallerian degeneration process might be impeded, and subsequent nerve regeneration might be affected. Furthermore, since the dynamic of actin cytoskeleton was shown to be related with cell survival (Sasi Kumar et al., 2015; Mattila et al., 2016), disruption of actin cytoskeleton might also impair nerve regeneration through inducing cell death. Additional experiments will be performed to test the role of cytochalasin D on multiple cell populations in the nerve stump to further determine the role of actin cytoskeleton in peripheral nerve regeneration.

Overall, by the joint use of RNA deep sequencing, IPA bioinformatic analysis and pharmacological treatment, our study demonstrated the implications of actin cytoskeleton following peripheral nerve injury from the molecular aspect, deepen the understanding of the underlying mechanisms of peripheral nerve injury, and might help the investigation of novel therapeutical targets of peripheral nerve repair and regeneration.

## Author contributions

SY conceived and designed the experiments. YW, QS, and SY performed the experiments. YW, QS, JP, and SY analyzed the data. SY contributed reagents, materials, analysis tools. YW and SY wrote the manuscript.

### Conflict of interest statement

The authors declare that the research was conducted in the absence of any commercial or financial relationships that could be construed as a potential conflict of interest.

## Supplementary material

Supplementary Material S1 qRT-PCR primer pair list.

Supplementary Material S2 IPA schematic diagram of actin cytoskeleton signaling at 1, 4, 7, and 14 days following sciatic nerve crush. Up-regulated genes were labeled in red while down-regulated genes were labeled in green.

Supplementary Material S3 Differentially expressed genes in actin cytoskeleton signaling at 1, 4, 7, and 14 days following sciatic nerve crush.

Supplementary Material S4 IPA schematic diagram of regulation of actin-based motility by Rho at 1, 4, 7, and 14 days following sciatic nerve crush. Up-regulated genes were labeled in red while down-regulated genes were labeled in green.

Supplementary Material S5 Differentially expressed genes in regulation of actin-based motility by Rho at 1, 4, 7, and 14 days following sciatic nerve crush.

Supplementary Material S6 Representative immunohistochemistry images of rat sciatic nerve treated with treated with 1000 nM cytochalasin D at 5 days after sciatic nerve crush. Areas between the dotted lines indicated injury site. Red indicated S100 staining of Schwann cells, green indicated NF-H staining of axons, and blue indicated Hoechst staining of cell nuclei. Higher magnifications of boxed areas were shown in **(a)**. Arrows indicated the migrated Schwann cells. Scale bars represented 1,000 μm (above) and 20 μm (below).

Supplementary Material S7 The expression levels of actin cytoskeleton-related genes in the dorsal root ganglia at 1, 4, and 7 days following sciatic nerve crush. Up-regulated genes were labeled in red while down-regulated genes were labeled in green.
