# Supplementary figures and images for: Actin Cytoskeleton Affects Schwann Cell Migration and Peripheral Nerve Regeneration
Source: Front Physiol. 2018 Jan 25;9:23. doi: 10.3389/fphys.2018.00023 (PMC5788963; doi:10.3389/fphys.2018.00023)

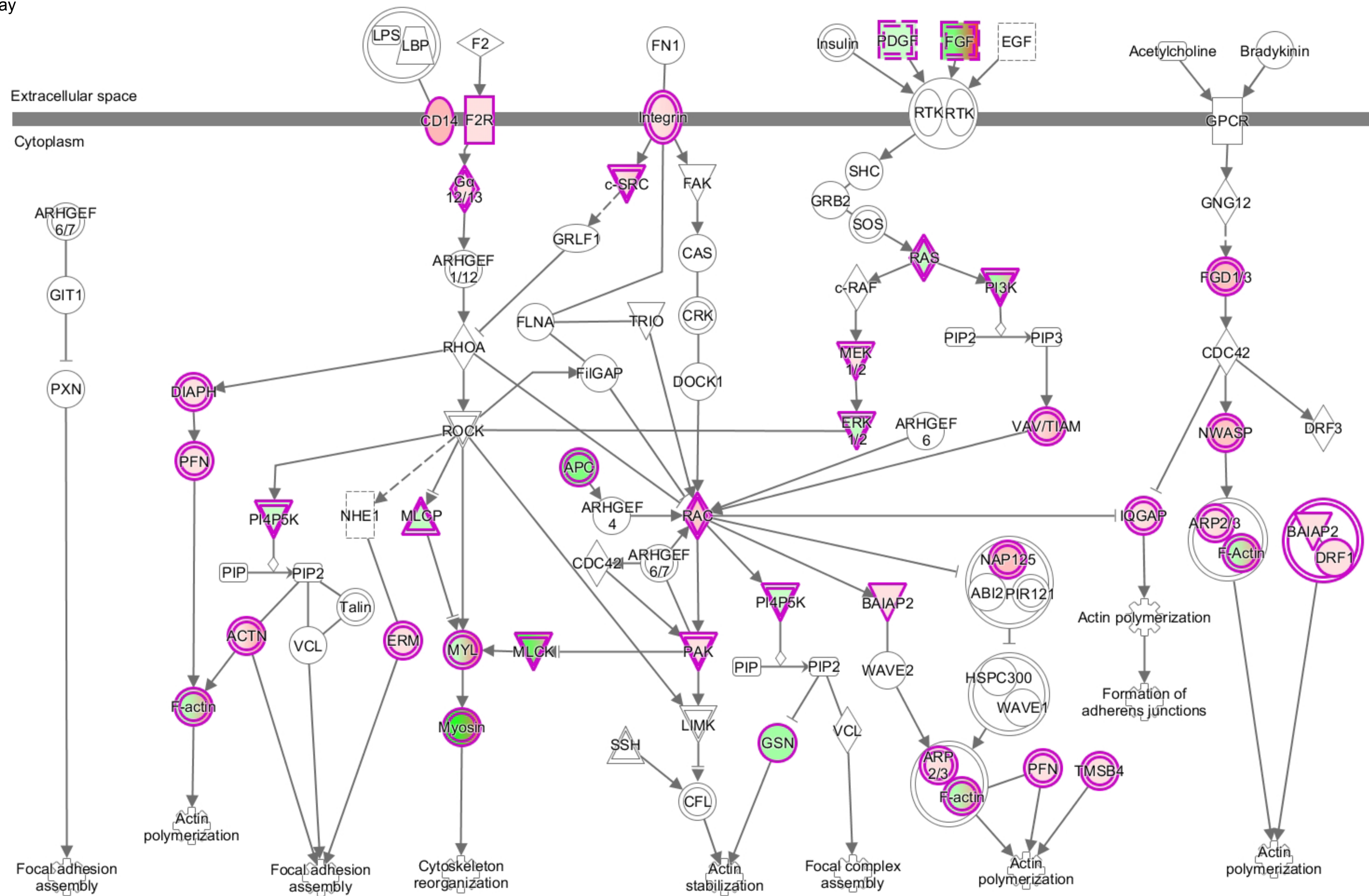

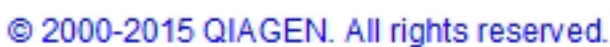

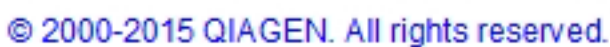

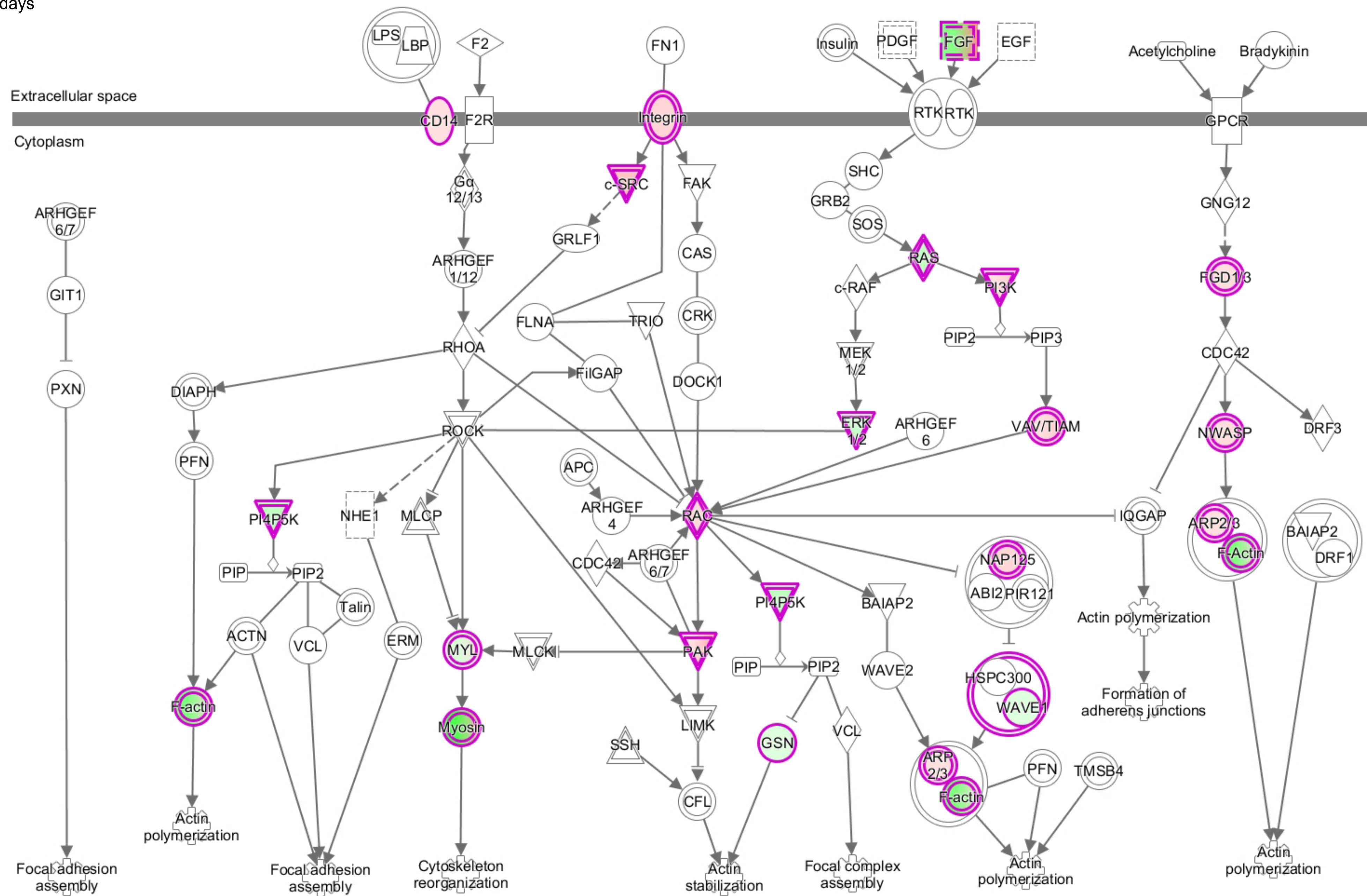

Supplement: Supplementary Material S2 — IPA schematic diagram of actin cytoskeleton signaling at 1, 4, 7, and 14 days following sciatic nerve crush. Up-regulated genes were labeled in red while down-regulated genes were labeled in green. [file DataSheet2.PDF]

1 day

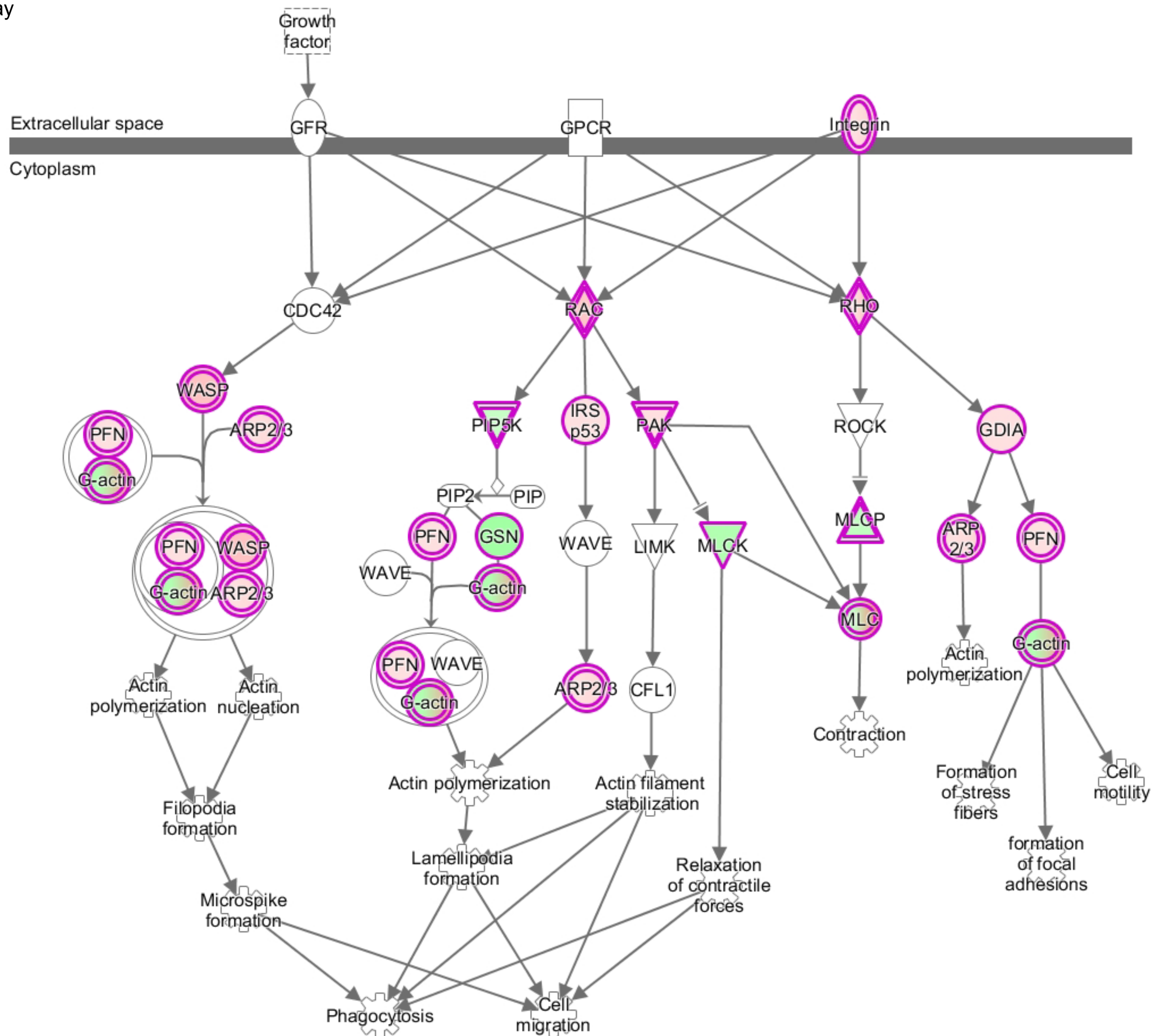

4 days

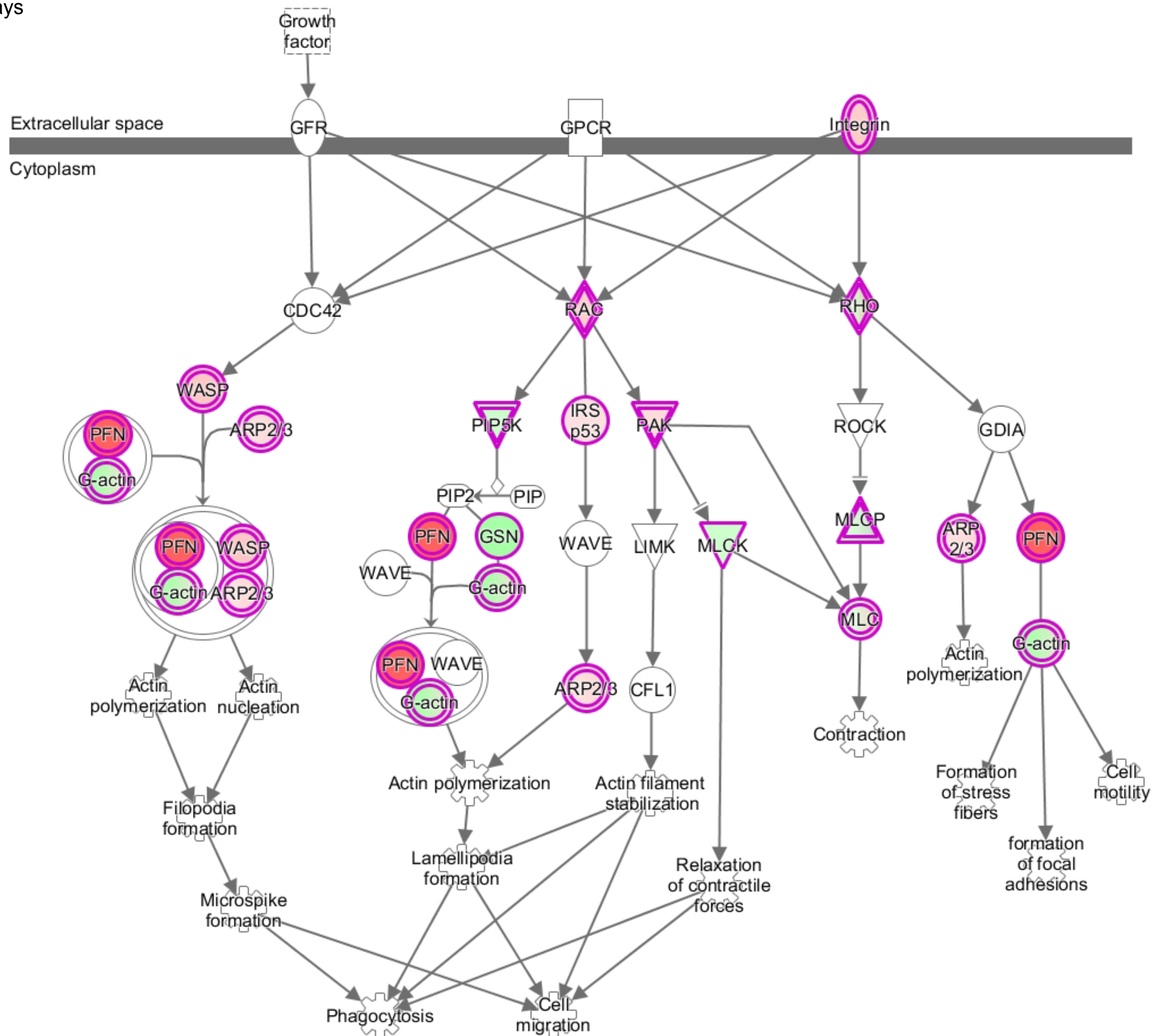

7 days

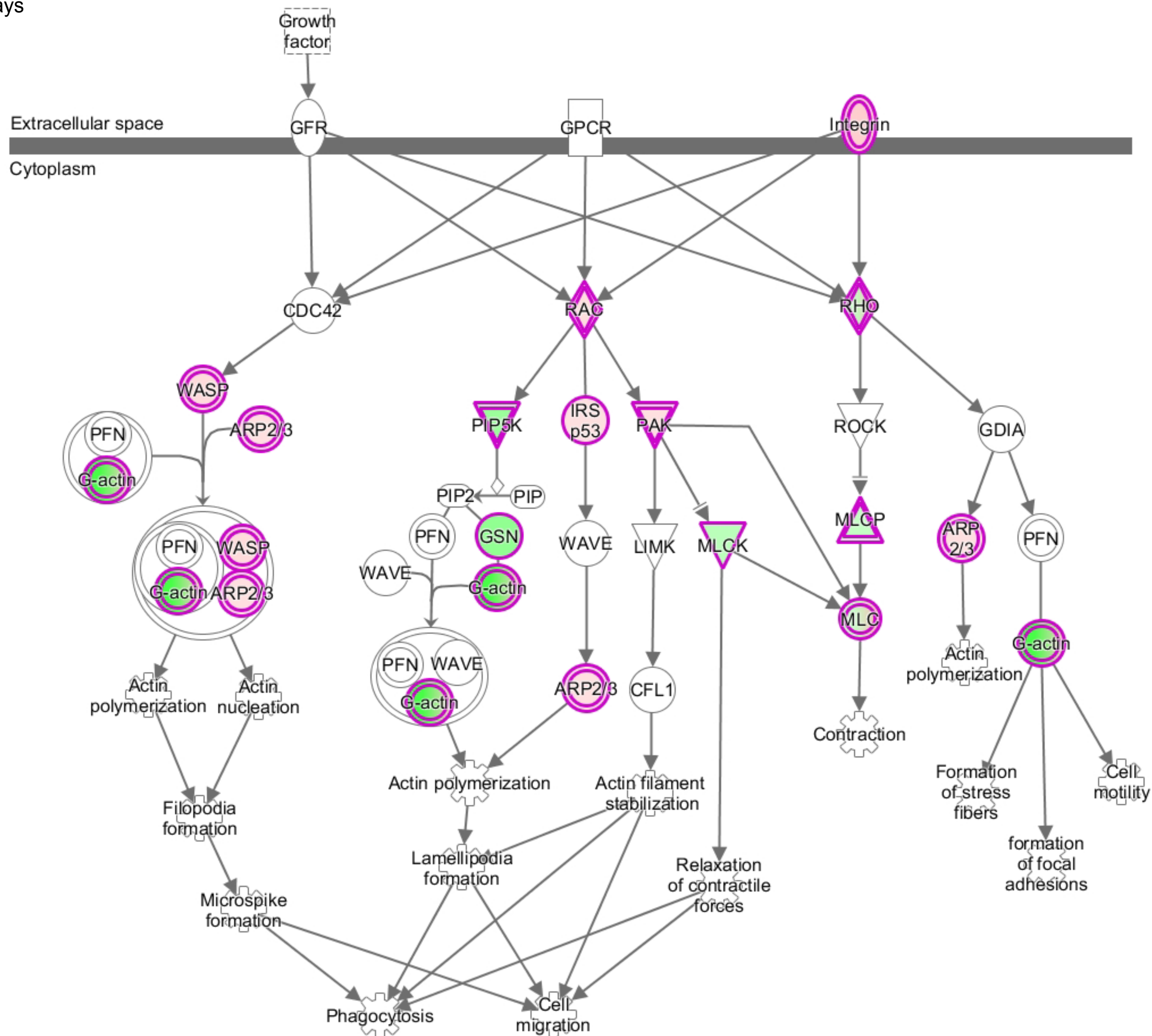

14 days

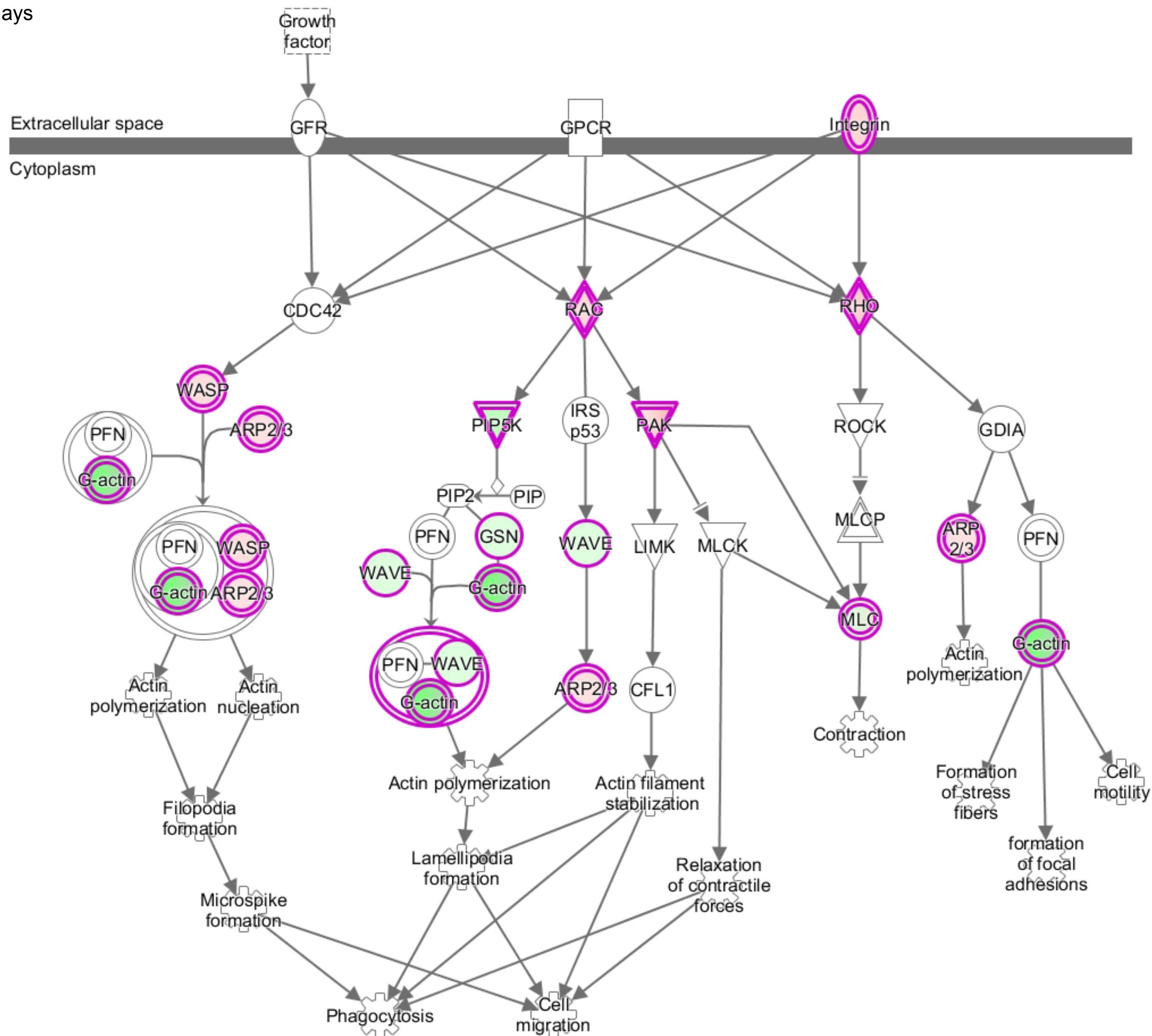

Supplement: Supplementary Material S4 — IPA schematic diagram of regulation of actin-based motility by Rho at 1, 4, 7, and 14 days following sciatic nerve crush. Up-regulated genes were labeled in red while down-regulated genes were labeled in green. [file DataSheet4.PDF]

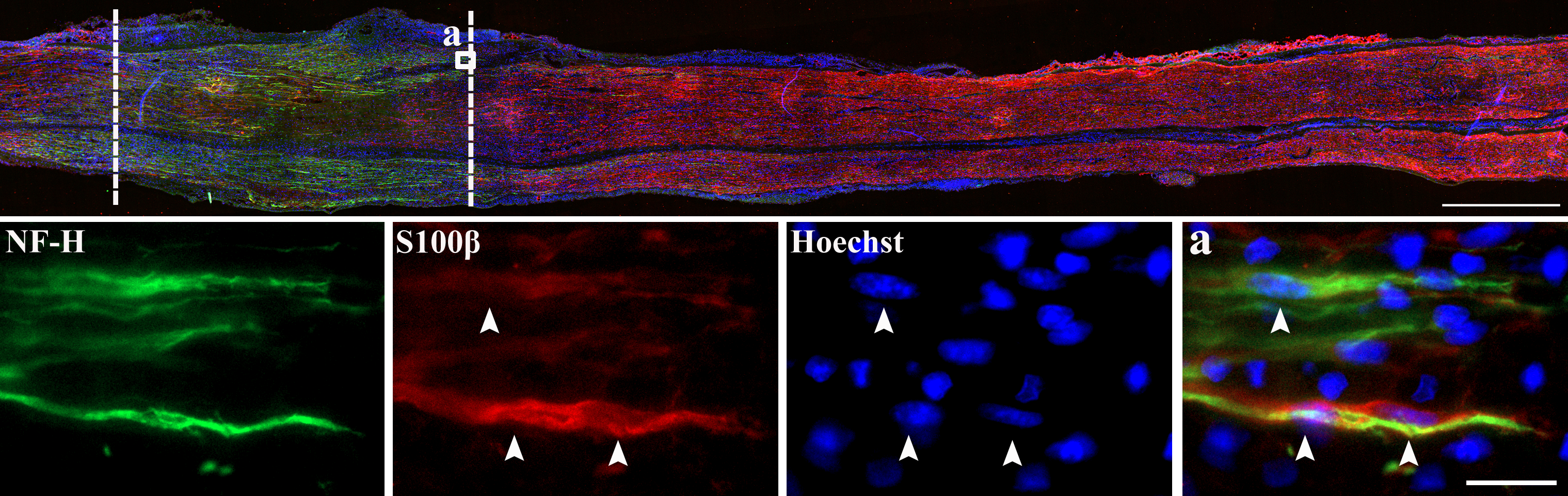

Supplement: Supplementary Material S6 — Representative immunohistochemistry images of rat sciatic nerve treated with treated with 1000 nM cytochalasin D at 5 days after sciatic nerve crush. Areas between the dotted lines indicated injury site. Red indicated S100 staining of Schwann cells, green indicated NF-H staining of axons, and blue indicated Hoechst staining of cell nuclei. Higher magnifications of boxed areas were shown in (a). Arrows indicated the migrated Schwann cells. Scale bars represented 1,000 μm (above) and 20 μm (below). [file DataSheet6.TIF]
